# Hot spring frogs (*Buergeria japonica*) prefer cooler water to hot water

**DOI:** 10.1002/ece3.6637

**Published:** 2020-08-11

**Authors:** Shohei Komaki, Yoichi Sutoh, Kensuke Kobayashi, Shigeru Saito, Claire T. Saito, Takeshi Igawa, Quintin Lau

**Affiliations:** ^1^ Division of Biomedical Information Analysis Iwate Tohoku Medical Megabank Organization Disaster Reconstruction Center Iwate Medical University Yahaba Japan; ^2^ Seisen Junior High School Kamakura Japan; ^3^ Division of Cell Signaling National Institute for Physiological Sciences National Institutes of Natural Sciences Okazaki Japan; ^4^ Amphibian Research Center Hiroshima University Higashi‐Hiroshima Japan; ^5^ Department of Evolutionary Studies of Biosystems Sokendai (The Graduate University for Advanced Studies) Hayama Japan

**Keywords:** Amphibian, behavioral observations, Rhacophoridae, thermal ecology

## Abstract

“Hot spring frog” is an informal name used for the Japanese stream tree frog (*Buergeria japonica*), which is widely distributed in Taiwan and the Ryukyu Archipelago in Japan. Some populations of the species are known to inhabit hot springs. However, water temperature can be extremely high around the sources of hot springs. Thus, it is questionable whether *B. japonica* selectively inhabits such dangerous environments. To address this question, we conducted a series of observations of water temperature preferences of a hot spring population of *B. japonica* in Kuchinoshima Island in Japan: (a) a field observation of tadpole density in water pools of different temperatures, (b) a field observation of water temperatures where adult males appear for breeding, and (c) an indoor observation of water temperatures selected by adult females for oviposition. As a result, tadpoles showed a higher density in cooler water. Adult males avoided water pools hotter than 37°C, and adult females selected cooler pools for oviposition. Camera records also showed that adult individuals tend to appear around cooler pools. Thus, we did not find any support for the hypothesis that hot spring frogs prefer hot water. Conversely, they apparently tended to prefer cooler water if it was available. Water temperatures around the sources of the hot spring exceed thermal tolerances of the species and could be a strong selective pressure on the population. Thus, the ability to sense and avoid lethal temperatures may be a key ecological and physiological characteristic for the species that inhabit hot springs.

## Graphical abstract

Hot spring frog (*Buergeria japonica*) inhabits hot springs and exhibits high thermal tolerance. Although, behavioral observations showed that they do not prefer hot waters. Our study suggests that ability to sense and avoid lethal temperatures are key ecological and physiological characteristics for the species that inhabit hot springs.

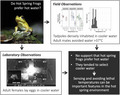

## 1 INTRODUCTION

The Japanese stream tree frog (*Buergeria japonica*) is widely distributed across Taiwan and the Ryukyu Archipelago in southern Japan. This frog is informally called the “hot spring frog" because it was initially reported to inhabit the Seranma hot spring in Kuchinoshima Island, Japan (Morita, 1988). In addition, *B. japonica* and a newly described sister species, *Buergeria otai* (Wang et al., 2017), have been found in Jentse, Rushan, and Wulai hot springs. Tadpoles of these species could tolerate a maximum temperature of 43–44°C (Chen, Kam, & Lin, 2001; Wu & Kam, 2005). A similar thermal tolerance was found in other *B. japonica* and *B. otai* tadpole populations in Taiwan (Yilan and Hualien) and the Ryukyu Archipelago (Amamioshima and Tokashikijima Islands) (Komaki, Igawa, Lin, & Sumida, 2016). Furthermore, Komaki, Lau, and Igawa (2016) performed field observations and physiological measurements of *B. japonica* tadpoles in the Seranma hot spring in Kuchinoshima Island and reported tadpoles inhabiting hot spring pools of up to 46.1°C in the wild and experimentally determined that tadpoles have a thermal limit of 46.2°C. This is the highest ever recorded water temperature inhabited by any frog population worldwide.

Ecology of amphibians in hot springs is scarcely reported to date, but several amphibian species outside of hot springs have been reported to select pools with a higher temperature water (Bancroft, Baker, Searle, Garcia, & Blaustein, 2008), and it is expected that there are benefits to inhabiting hot waters such as acceleration of growth rate and prevention of infectious diseases. For instance, the growth rates of several *Rana* tadpoles were positively correlated with water temperature (Sype, 1974), and the prevalence of *Batrachochytrium dendrobatidis* (*Bd*), the fungus responsible for causing chytridiomycosis in amphibians, decreased in hotter waters (Forrest & Schlaepfer, 2011), although *Bd* infection did not alter the thermal preference of host individuals (Sauer et al., 2018). *Buergeria japonica* inhabiting hot springs may also benefit from faster growth and reduced infectious pathogens, and this could be a contributing factor for this species inhabiting the Seranma hot spring in Kuchinoshima Island.

Kuchinoshima Island is a part of the Tokara Islands, which are located in the northern region of the Ryukyu Archipelago and represents the northern most natural distribution area of *B. japonica*. The Tokara Islands are small‐sized volcanic islands and are unique because freshwater sources, historically, have been scarce here. Therefore, the hot spring may provide an important habitat for any frog species on these islands. *Buergeria japonica* is the only endemic frog species that inhabits the Tokara Islands (Maenosono & Toda, 2007). Thus, it is likely that the ability to use the hot springs and tolerate the high water temperatures has been beneficial for the survival of this species on the Tokara Islands.

**Figure 1:**
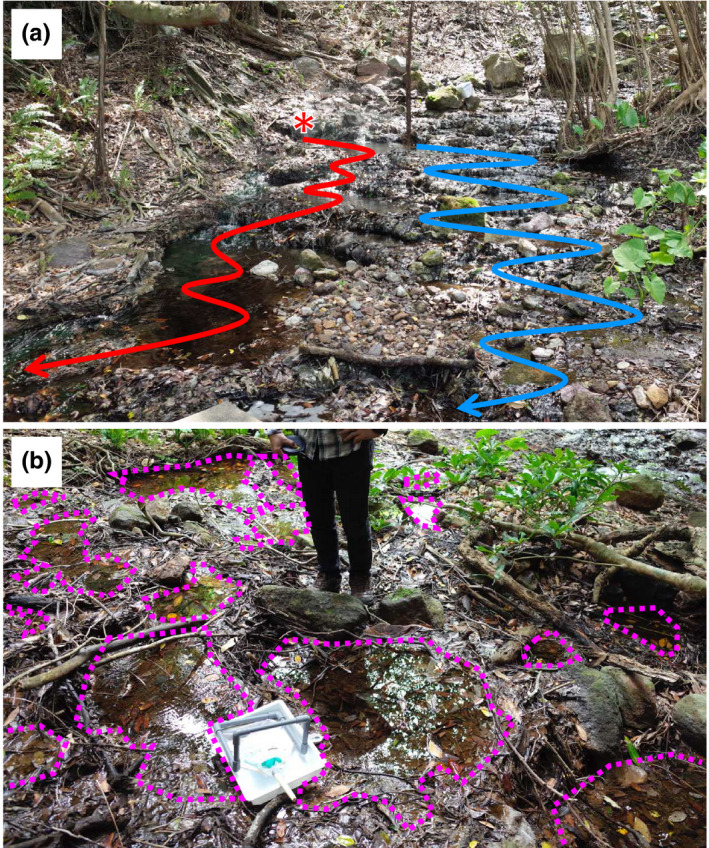
A study site in the Seranma hot spring. (a) An overview of water flow. A stream on the left side, where water flowed directly from a source of the hot spring (indicated by the asterisk), showed higher temperature (>50°C), whereas a shallow stream on the right side showed lower water temperature. There were multiple water paths connecting the two streams where hot and cool water exchanged. The photograph was taken on September 9, 2015. (b) Water pools formed along the streams (circled by magenta dashed lines). Each pool was dammed by fallen leaves, branches, or stones, although water still flowed between pools

Despite hot springs being an apparently essential habitat for *B. japonica*, hot spring habitats have some areas with dangerously high temperatures (Figure 1a). Wu and Kam (2005) showed that tadpoles avoided water above 37°C in both the field and laboratory. Our observations together with their findings raise a question about whether *B. japonica* selectively inhabits hot springs; it is essential to address this question in order to understand their unusual ecological characteristics.

*Buergeria japonica* tadpoles are completely aquatic, and thus, their occurrence in hot springs is expected to be strongly affected by the oviposition site selected by adults. Therefore, in this study, through behavioral observations of tadpoles and adults, we tested the hypothesis that *B. japonica* prefer hot water. We aimed to clarify if *B. japonica* inhabits hot spring due to thermal preference for hot water.

## 2 MATERIALS AND METHODS

### 2.1 Study area and seasons

The Seranma hot spring is a geothermal hot spring located on Kuchinoshima Island, Toshima Village, Kagoshima Prefecture, Japan (29°57.32′N, 129°55.63′E). The hot spring is an assembly of shallow and narrow streams (Figure 1a). There is a pedestrian bridge connecting hot spring facilities across streams, and we set the study area upstream of the bridge because drainage from the man‐made facility flowed into the stream beneath the bridge, which can affect quantity, quality, and temperature of water. The width of the hot spring in the study area including two major streams is <10 m and the length was approximately 100 m.

Ecology of organisms in the Tokara Islands has been scarcely studied. The breeding season of *B. japonica* ranges between March and November across the entire distribution that includes tropical rainforest and humid subtropical climates (Matsui & Maeda, 2018), and we expect that the season is much narrower in the populations in the Tokara Islands, which is the northern edge of the species distribution. Therefore, for collection of data, we performed fieldwork during active and breeding seasons of *B. japonica* in spring and summer.

### 2.2 Tadpole density surveys

**Figure 2:**
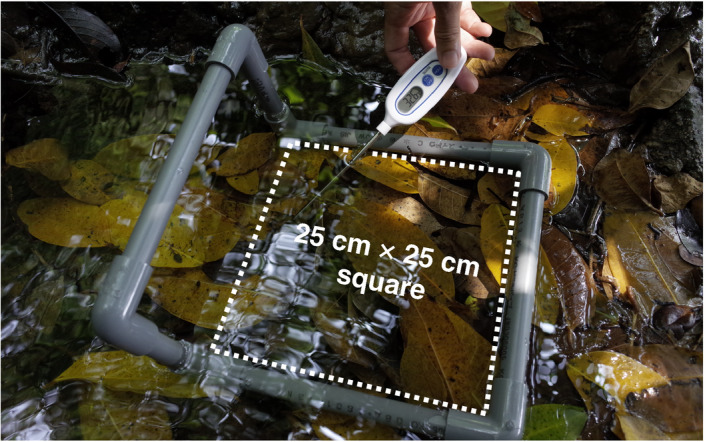
Survey of tadpole densities. Tadpole density was measured within a 25 cm × 25 cm square frame. Imitating a Surber sampler, a net bag was placed on one side of the frame (left side in the picture) and all litter was quickly pushed into the net bag to collect all tadpoles inside the frame. Water temperatures at one or more points within the square were measured to analyze the relationship between water temperature and tadpole density

To infer the water temperature preference of tadpoles, we measured the density of tadpoles in water pools in the Seranma hot spring in Kuchinoshima Island across three sampling periods: April 17–21 in 2018 (*n* = 12), August 7–9 in 2018 (*n* = 17), and April 9–13 in 2019 (*n* = 25). All measurements were conducted between 9 a.m. and 4 p.m. To quantify the density of tadpoles, we used a 25 cm × 25 cm square frame for the measurements (Figure 2). First, we measured water temperature of one or two randomly selected points at the bottom of the square. For the collection of tadpoles, based on initial trials, we applied a method imitating a Surber sampler to prevent tadpole escape; we placed a D‐frame dip net bag on one side of the frame (Malard, Dole‐Olivier, Mathieu, & Stoch, 2002) and quickly pushed all litter into the net bag. After collection, we spread the litter on a plastic laboratory tray and sorted out and counted the tadpoles. In a previous study, the highest water temperature where a tadpole was observed was 46°C (Komaki, Lau, et al., 2016). Thus, we only used study sites with water pool temperatures of 46°C or lower. To measure the water temperature, we used Tanita Digital Thermometers (TT‐508‐WH; Tanita Corporation).

The relationship between tadpole density and water temperature was analyzed using a simple linear regression model and the Wilcoxon rank sum test. The count of tadpoles included some extreme values that influence the linear regression analysis. To account for these extreme values, we used log_10_‐transformed counts of tadpoles, which made the data more normally distributed (e.g., Helsen et al., 2017; Verberk, Velde, & Esselink, 2010). In the regression analyses, the log‐transformed tadpole count and water temperature were used as the dependent and explanatory variables, respectively. The analyses were independently performed for the three datasets (April 2018, August 2018, and April 2019) and a combined dataset of April 2018 and 2019. In the Wilcoxon rank sum test, we divided water temperatures into the following four categories: <30°C, 30–35°C, 35–40°C, and ≥40°C, and compared one category at a time with all the other groups pooled as one group. The tests were independently performed for the three datasets and a combined dataset using the package “exactRankTests” (Hothorn & Hornik, 2019) in R 3.6.3 (R Core Team, 2020). All statistical procedures were carried out using R 3.6.3.

### 2.3 Observations of temperature selection of adult males

To investigate the water temperature preference of adult males for a breeding site, we measured the temperature of water where adult males were found at night (8–11 p.m.) in the Seranma hot spring during May 27–30 in 2017 and April 17–21 in 2018. Only males found inside or at the edge of water bodies (<20 cm away from the water body) and expressing the breeding color of bright yellow were considered. To measure the temperature, we used Tanita Digital Thermometers.

### 2.4 Indoor observations of oviposition behavior

To investigate the water temperature preferences of adult frogs for oviposition, we initially performed preliminary field observation of oviposition behavior through visual observation or by using infrared cameras. However, there was an insufficient number of amplexus pairs observed and insufficient resolution of the infrared cameras. Therefore, we conducted three indoor behavioral observation experiments during April 27–29 and August 8–10 in 2018. We collected adult females and males from the Seranma hot spring, and released them into a net cage (mosquito net; L 115 cm × W 85 cm × H 85 cm) for one or two nights. In the cage, we placed three water pools (L 11.7 cm × W 15.6 cm × H 5.3 cm) with temperatures maintained at 25, 30, and 35°C, respectively. Water temperatures were maintained by heaters (Nisso Power Safe Heater; Marukan Co. Ltd). Heater controllers (REI‐SEA TC‐101; Iwaki Co., Ltd and NX003N; GEX Corporation) were used to prevent overheating. Every morning during the observation period, we counted the number of spawned egg clutches in each pool. In each experiment, approximately 10 females and 10 males were released into the cage. The behavior of frogs was recorded by taking photos every 2 min using an infrared camera during the experiment conducted on August 8–10.

## 3 RESULTS

### 3.1 Characteristics of the study site

Along the hot spring streams, chains of small shallow pools (approximately 100 pools, mostly <1 m in diameter and <10 cm in depth) were disorderly formed by fallen leaves, branches, or stones that dammed up the water (Figure 1b). There were at least four sources of hot spring from which all water pools were derived from, and water paths converged within the study area. The major source of the spring was on the left side of the stream; therefore, water temperature was higher in that side of the stream where hot water directly flows through (Figure 1a). On the right side of the stream, shallower water pools were formed, of which water temperature was lower. Water temperatures of these hot and cool streams had a range of approximately 50–75°C and 20–35°C, respectively, and were almost stable during surveys of tadpole density and adult males. In contrast, the water path was intricate, and the hot and cool streams exchanged water in some pools that resulted in variation of water temperature among pools (approximate range: 30–50°C). Because the hot and cool streams were parallel to each other in a narrow study area, there was a drastic horizontal gradient of water temperature. Some cool water pools (<30°C) were found within 10 m from the source of the hot spring (>75°C), although they were not directly connected to each other by a water path.

Locations of the hot spring sources were unchanged for at least 5 years since the previous study in this area (Komaki, Lau, et al., 2016); therefore, the major spatial gradient of water temperature in the study area seems to be stable. Conversely, water temperatures in pools where hot and cool water meets were relatively unstable: Even just a fallen leaf stuck in the water path could change water flow that can result in the fluctuation of water temperature of the downstream pools. Although not experimentally tested, we expect that temperatures of small water pools can quickly exceed 40°C when diverted hot stream water flow into them.

It was difficult for tadpoles to migrate to upstream water pools because small cascades were formed between upstream and downstream pools. However, it was possible for tadpoles to move horizontally between neighboring pools or downstream along the water path. Therefore, tadpoles could move into desirable thermal environments if they have thermal preference. Similar to previous observations in the Seranma hot spring (Komaki, Lau, et al., 2016), tadpoles were found in water pools with temperature up to 46°C.

In the study area, there was no stable water source other than the hot spring. Moreover, we found no *B. japonica* individuals outside of the hot spring region (>500 m away from water body). Therefore, seemingly, the *B. japonica* population in the Seranma hot spring is relatively isolated and relies heavily on water resources of this hot spring.

### 3.2 Tadpole density

**Figure 3:**
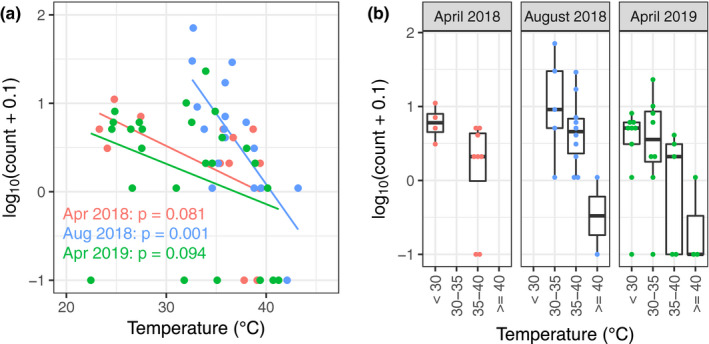
Relationships between the mean water temperature and density of tadpoles. (a) A plot with linear regression lines for the three datasets. *p* values from linear regression analyses are also presented. (b) A boxplot of categorized datasets. The surveys were conducted in April 2018, August 2018, and April 2019, and plotted independently (red, blue, and green, respectively). Within our study area, there were no water pools below 30°C in August

**Figure 4:**
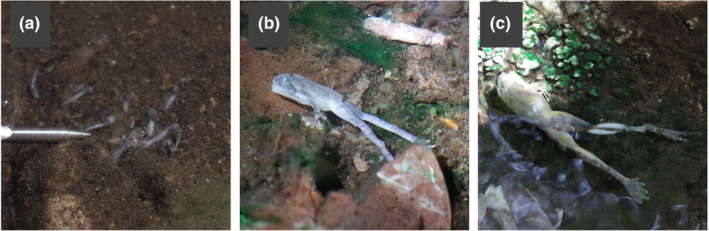
Dead tadpoles (a), a juvenile (b), and an adult of *Buergeria japonica* (c) found in the Seranma hot spring. Water temperatures were above 45°C

In this survey, we measured tadpole densities at 54 sites. The tadpole count in the pools ranged from 0 to 71 (median of 3.5). From the linear regression analyses, a trend where tadpole densities decreased with higher temperatures was found. This trend was statistically significant in the August 2018 data and the combined dataset of April 2018 and April 2019 (*p* < .05; Table 1 and Figure 3a). The Wilcoxon rank sum test showed that the tadpole density at <30°C was significantly higher than that at 35–40°C in April 2018 and the density at >40°C was significantly lower than that at the other temperature categories in both August 2018 and April 2019 (Figure 3b and Table 2). During the survey, we also found dead marten, lizards, land snails, and caterpillars, as well as dead *B. japonica* in the spring water pools above 50°C (Figure 4). In the litter collected from 25°C water pools, live dragonfly larvae were also found.

**Table 1:** Results of linear regression analyses of tadpole density. The density was measured by counting tadpoles collected inside a 25 cm × 25 cm square frame. Then, log‐transformed density was regressed on water temperature (°C). Results with *p* value < .05 are shown in bold font. *SE*, standard error

Dataset	Coefficient (*SE*)	*p* value
April 2018	−0.055 (0.032)	.081
August 2018	**−0.160 (0.043)**	**.001**
April 2019	−0.046 (0.028)	.094
April (2018 and 2019, combined)	**−0.048 (0.019)**	**.018**

**Table 2:** Results of the Wilcoxon rank sum tests of tadpole density. Comparisons of results with *p* value < .05 are shown in bold font. Some comparisons were not analyzed (NA) because data in certain temperature categories were not collected in given seasons

Dataset	Comparison			
	<30°C and other	30–35°C and other	35–40°C and other	≥40°C and other
April 2018	**0.044**	NA	**0.044**	NA
August 2018	NA	0.149	0.982	**0.044**
April 2019	0.229	0.228	0.220	**0.047**
April (combined)	**0.026**	0.327	0.063	**0.033**

### 3.3 Distribution of adult males

**Figure 5:**
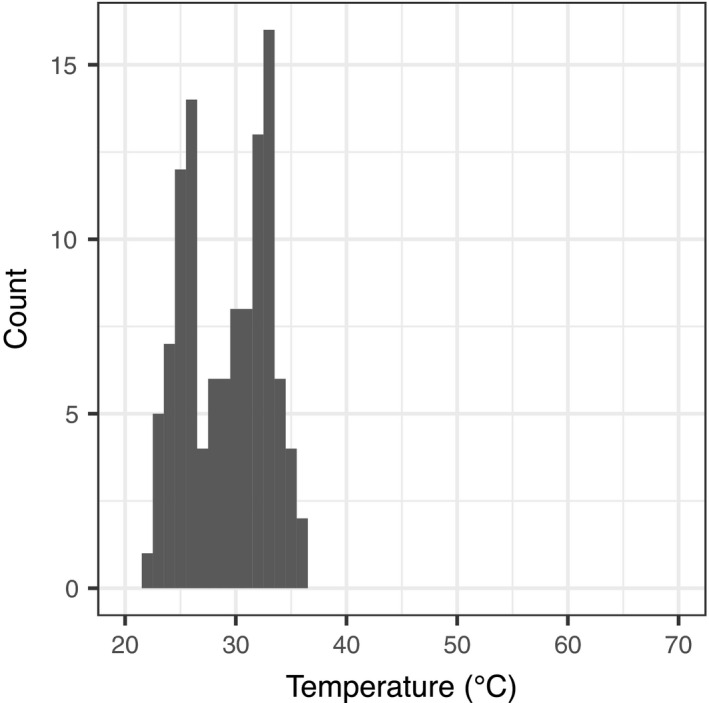
Number of adult males found at each water temperature in a breeding site. The surveys were performed during May 27–30 in 2017 and April 17–21 in 2018 in the Seranma hot spring. Only males expressing the breeding color of bright yellow were considered. The width of bins in the histogram is 1°C

In the survey of adult males, we found 112 males inside or at the edge of water bodies. We did not find any male individuals in and around water pools hotter than 37°C (Figure 5). The distribution of adult males showed two peaks, but we did not measure the density of individuals. The water temperature of most pools was 30°C or higher.

### 3.4 Thermal selection for oviposition

**Figure 6:**
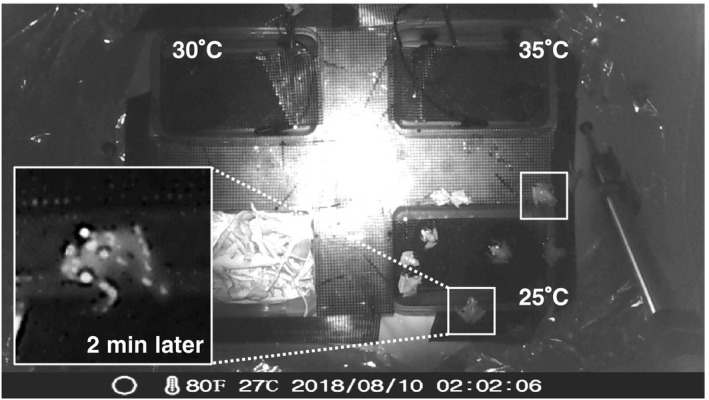
Infrared image taken during the breeding experiment. Individuals surrounded by white squares were pairs in amplexus. Three pools with different water temperatures and a tank filled with leaves (left bottom) were placed in the cage and the frogs were photographed every 2 min to monitor their water temperature preferences. An enlarged picture shows an amplexus pair after 2 min (02:04:06) holding the edge of a pool and seemingly ovipositing

**Figure 7:**
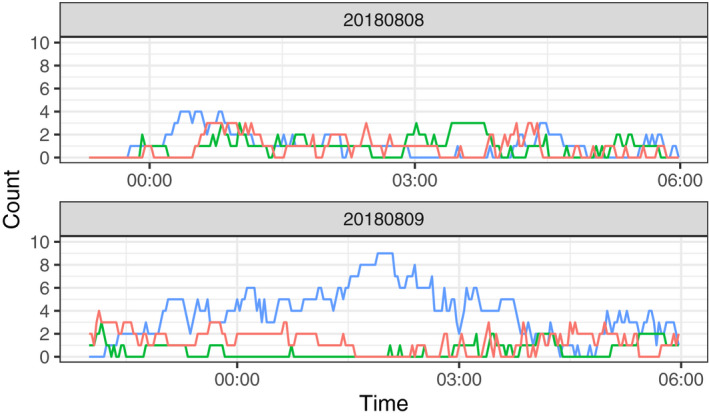
Number of individuals found in water pools with different temperatures during the indoor observations (blue: 25°C, green: 30°C, red: 35°C). In the top panel, data were collected from 23:19 on 2018/08/08 to 6:00 on 2018/08/09 and the data in the bottom panel were collected from 22:00 on 2018/08/09 to 6:00 on 2018/08/10. Any amplexed pair was counted as one individual

**Figure 8:**
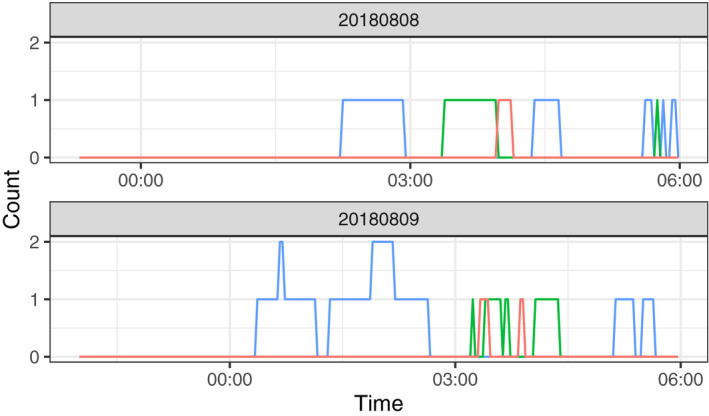
Number of amplexed pairs found in water pools of different temperatures during the indoor observations (blue: 25°C, green: 30°C, red: 35°C). In the top panel, data were collected from 23:19 on 2018/08/08 to 6:00 on 2018/08/09; the data in the bottom panel were collected from 22:00 on 2018/08/09 to 6:00 on 2018/08/10

In the experiment to determine female oviposition preference, we observed seven oviposition events, among which five were in 25°C water pools and two were in 30°C water pools (Table 3). No eggs were laid in the 35°C water pool, although more than two pairs were temporarily observed in this pool based on camera records. In each event, more than 100 eggs were laid except for that in the 30°C water pool on August 9, 2018, whereby approximately 50 eggs were laid. According to the infrared camera records on August 9, the 25°C water pool showed the highest density of individuals and amplexus pairs (Figures 6, 7, 8).

**Table 3:** Total number of egg clutches laid in the pools of different water temperatures during the indoor observations. The observations were conducted during April 27–29 and August 8–9 in 2018

Water temperature	Egg clutch count (Date of oviposition)
25°C	5 (Apr. 27th × 1, 28th × 1, 29th × 1, Aug. 9th × 2)
30°C	2 (Apr. 27th × 1, Aug. 9th × 1)
35°C	0

## 4 DISCUSSION

For our tadpole density survey, it should be noted that the sample size was small as the study site is an assembly of small streams, which limited the number of surveys. Nevertheless, our results suggested that lower temperature water pools were more densely inhabited by tadpoles than hot water pools. We cannot deny that the trend of tadpole density was affected by (a) adult females’ choice of lower temperatures for oviposition, which was observed in this study, or (b) the habitat characteristics whereby water temperature of pools formed between hot and cool streams can fluctuate due to changes in water path and tadpoles in such pools died due to the sudden temperature spike, which can result in lower tadpole densities in hotter pools. Nevertheless, tadpoles were able to choose water temperatures by moving downstream or horizontally between neighboring pools, and the results are consistent with a previous study in which *B. otai* tadpoles of a hot spring population showed preference for cooler water in a laboratory experiment (Wu & Kam, 2005). Therefore, we expect that the result of our tadpole density survey, at least partially, reflects the thermal preference of *B. japonica* tadpoles.

In our field observations, adult males avoided hot water. The maximum water temperature where adult males were found was 36.2°C. In our indoor observations, the 25°C water pool showed the highest density of individuals of both sexes, although factors other than the water temperature, such as mating calls and existence of individuals around the pool, could have attracted further individuals and enhanced the density. The distribution of water temperature where adult males were found had two peaks around 25 and 33°C. We did not measure the density of adult males, and therefore it remains unclear whether this bimodal distribution reflects the thermal preference of adult males. However, this finding may be largely attributed to habitat characteristics; according to the records collected in our tadpole density survey, distribution of water temperature in the study site was also bimodal with the highest and second highest peaks around 36 and 26°C, respectively. Moreover, adult males showed preference for 25°C water pool rather than 30°C or 35°C water pools according to our indoor observation.

Similarly, females tended to select cooler water pools for oviposition. We could not conduct field observations of oviposition, and therefore, it remains unclear if females lay eggs in hot water pools if only such pools are available around them. Nevertheless, our results showed that females do not lay eggs in hotter waters if they can choose cooler pools. The amplexus pairs temporarily resided in the 35°C water pool without laying eggs but spent the majority of their time and oviposited in the 25°C pool, supporting that frogs displayed behaviors of selecting preferable water temperatures for egg laying. Furthermore, European water frog females were reported to reduce their clutch size in response to unpreferable conditions (i.e., amplexed by undesired males, Reyer, Frei, & Som, 1999). In our study, we observed a reduced clutch size in a 30°C water pool in August and this may also be a result of maternal manipulation.

Our findings showed that despite commonly using hot spring environments, it appears that *B. japonica* in Kuchinoshima Island, known as the “hot spring frog,” still prefer to use and oviposit in cooler water pools. Thus, habitation and breeding in a hot spring environment is a unique characteristic among amphibians, but this feature may not be a result of the thermal preference of *B. japonica* for hot water. Instead, it may be possible that the utilization of hot springs is a product of inhabiting a tiny volcanic island whereby freshwater resources were historically limited.

*Buergeria japonica* in the Seranma hot spring population exhibited high thermal tolerance (Komaki, Lau, et al., 2016); however, this does not seem to be the result of adaptation to the hot spring environment. Because thermal tolerance is retained across their wide distribution range (Komaki, Igawa, et al., 2016) and it was shown that *B. japonica* have expanded northward in their distribution from Taiwan and that Kuchinoshima Island is one of their most recently established habitats (Komaki et al., 2017). Therefore, their thermal tolerance must have been acquired before the hot spring population in Kuchinoshima Island was established. Several anuran species exhibit high thermal tolerance, although only *B. japonica* and *B. otai* are known to inhabit and reproduce in hot springs. This may be because there are ecological, physiological, and behavioral factors other than thermal tolerance for coping with the extreme environment, as suggested by Wu and Kam (2005). Our study emphasizes the importance of the ability to sense temperature and avoid lethal temperatures when inhabiting a hot spring environment. Some water pools in the Seranma hot spring exhibited lethal temperatures of up to 70°C, and thus, sensing and avoiding such hot waters would be crucial for the survival of individuals. Specifically, reproductive behaviors of adult males and females such as avoiding hot water should reduce mortality of adults due to lethal temperatures and also reduce the risk of offspring being exposed to lethal water temperatures. However, temperature in water pools can fluctuate due to changes in water path; thus, avoiding hot water may also be an important behavioral trait in tadpoles for surviving in the hot spring area. Therefore, for tadpole and adult *B. japonica*, there must be an ongoing selective pressure on sensing and avoiding lethal water temperatures, and this could be a future research focus for clarifying how anuran populations adapt to extreme environments.

Finally, we note that our study did not address how air temperature can affect their preference of water temperature. In particular, cooler air temperature may affect the preference of adult individuals. Future laboratory experiments with lower air temperature will clarify if *B. japonica* prefer hotter water under cooler conditions or if they show stronger preference for cooler water to minimize the temperature differences between body and water temperatures.

## CONFLICT OF INTEREST

Authors have no conflict of interest to declare.

## AUTHOR CONTRIBUTIONS

**Shohei Komaki:** Conceptualization (lead); Data curation (lead); Formal analysis (lead); Funding acquisition (equal); Investigation (equal); Methodology (equal); Project administration (lead); Visualization (lead); Writing‐original draft (lead); Writing‐review & editing (lead). **Yoichi Sutoh:** Conceptualization (supporting); Investigation (equal); Methodology (equal); Visualization (supporting); Writing‐original draft (supporting); Writing‐review & editing (equal). **Kensuke Kobayashi:** Conceptualization (supporting); Investigation (equal); Methodology (equal); Visualization (supporting); Writing‐original draft (supporting); Writing‐review & editing (equal). **Shigeru Saito:** Conceptualization (supporting); Funding acquisition (equal); Investigation (equal); Methodology (supporting); Visualization (supporting); Writing‐original draft (equal); Writing‐review & editing (equal). **Claire Saito:** Conceptualization (supporting); Investigation (equal); Methodology (supporting); Visualization (supporting); Writing‐original draft (equal); Writing‐review & editing (equal). **Takeshi Igawa:** Conceptualization (supporting); Funding acquisition (equal); Investigation (equal); Methodology (supporting); Visualization (supporting); Writing‐original draft (equal); Writing‐review & editing (equal). **Quintin Lau:** Conceptualization (supporting); Data curation (supporting); Formal analysis (supporting); Funding acquisition (equal); Investigation (equal); Methodology (equal); Visualization (supporting); Writing‐original draft (lead); Writing‐review & editing (equal).

## Data Availability

Data and scripts are available on Dryad (https://doi.org/10.5061/dryad.f1vhhmgtt).
